# Supplementary material for: HIVprotI: an integrated web based platform for prediction and design of HIV proteins inhibitors
Source: J Cheminform. 2018 Mar 9;10:12. doi: 10.1186/s13321-018-0266-y (PMC5845081; doi:10.1186/s13321-018-0266-y)
Supplement: Supplementary file 2 — Additional file 2. Source code of HIVProtI web server. [file 13321_2018_266_MOESM2_ESM.zip › HIVprotI_Source-code/hivprotI/weka-3-6-6/doc/weka/gui/beans/BatchClassifierEvent.html]

BatchClassifierEvent


---


|  |  |  |
| --- | --- | --- |
| **Overview** \| **Package** \| **Class** \| **Tree** \| **Deprecated** \| **Index** \| **Help** \| **Weka's home** | |  |
| **PREV CLASS**   **NEXT CLASS** | **FRAMES**    **NO FRAMES**     **All Classes** |  |
| SUMMARY: NESTED \| FIELD \| CONSTR \| METHOD | DETAIL: FIELD \| CONSTR \| METHOD |  |


---


## weka.gui.beans Class BatchClassifierEvent

```
java.lang.Object
  java.util.EventObject
      weka.gui.beans.BatchClassifierEvent
```

**All Implemented Interfaces:**: java.io.Serializable

---

``` public class BatchClassifierEvent extends java.util.EventObject ```

Class encapsulating a built classifier and a batch of instances to
test on.

**Since:**
:   1.0

**Version:**
:   $Revision: 5782 $

**Author:**
:   Mark Hall

**See Also:**: `EventObject`, Serialized Form

---

**Constructor Summary**

`BatchClassifierEvent(java.lang.Object source,
Classifier scheme,
DataSetEvent trsI,
DataSetEvent tstI,
int setNum,
int maxSetNum)`
  
          Creates a new `BatchClassifierEvent` instance.

`BatchClassifierEvent(java.lang.Object source,
Classifier scheme,
DataSetEvent trsI,
DataSetEvent tstI,
int runNum,
int maxRunNum,
int setNum,
int maxSetNum)`
  
          Creates a new `BatchClassifierEvent` instance.


| **Method Summary** | |
| --- | --- |
| `Classifier` | `getClassifier()`             Get the classifier |
| `long` | `getGroupIdentifier()` |
| `int` | `getMaxRunNumber()`             Get the maximum run number |
| `int` | `getMaxSetNumber()`             Get the maximum set number (ie the total number of training and testing sets in the series). |
| `int` | `getRunNumber()`             Get the run number. |
| `int` | `getSetNumber()`             Get the set number (ie which fold this is) |
| `DataSetEvent` | `getTestSet()`             Get the test set |
| `DataSetEvent` | `getTrainSet()`             Get the train set |
| `void` | `setClassifier(Classifier classifier)`             Set the classifier |
| `void` | `setGroupIdentifier(long identifier)` |
| `void` | `setTestSet(DataSetEvent tse)`             Set the test set |
| `void` | `setTrainSet(DataSetEvent tse)`             Set the training set |

**Methods inherited from class java.util.EventObject**

`getSource, toString`

**Methods inherited from class java.lang.Object**

`equals, getClass, hashCode, notify, notifyAll, wait, wait, wait`

**Constructor Detail**

### BatchClassifierEvent

```
public BatchClassifierEvent(java.lang.Object source,
                            Classifier scheme,
                            DataSetEvent trsI,
                            DataSetEvent tstI,
                            int setNum,
                            int maxSetNum)
```

:   Creates a new `BatchClassifierEvent` instance.

    **Parameters:**: `source` - the source object: `scheme` - a Classifier: `trsI` - the training instances used to train the classifier: `tstI` - the test instances: `setNum` - the set number of the test instances: `maxSetNum` - the last set number in the series

---


### BatchClassifierEvent

```
public BatchClassifierEvent(java.lang.Object source,
                            Classifier scheme,
                            DataSetEvent trsI,
                            DataSetEvent tstI,
                            int runNum,
                            int maxRunNum,
                            int setNum,
                            int maxSetNum)
```

:   Creates a new `BatchClassifierEvent` instance.

    **Parameters:**: `source` - the source object: `scheme` - a Classifier: `trsI` - the training instances used to train the classifier: `tstI` - the test instances: `runNum` - the run number: `maxRunNum` - the maximum run number: `setNum` - the set number of the test instances: `maxSetNum` - the last set number in the series


**Method Detail**

### getClassifier

```
public Classifier getClassifier()
```

:   Get the classifier

    :   **Returns:**: the classifier

---


### setClassifier

```
public void setClassifier(Classifier classifier)
```

:   Set the classifier

    :   **Parameters:**: `classifier` - the classifier

---


### setTestSet

```
public void setTestSet(DataSetEvent tse)
```

:   Set the test set

    :   **Parameters:**: `tse` - the test set

---


### getTestSet

```
public DataSetEvent getTestSet()
```

:   Get the test set

    :   **Returns:**: the test set

---


### setTrainSet

```
public void setTrainSet(DataSetEvent tse)
```

:   Set the training set

    :   **Parameters:**: `tse` - the training set

---


### getTrainSet

```
public DataSetEvent getTrainSet()
```

:   Get the train set

    :   **Returns:**: the training set

---


### getRunNumber

```
public int getRunNumber()
```

:   Get the run number.

    :   **Returns:**: the run number

---


### getMaxRunNumber

```
public int getMaxRunNumber()
```

:   Get the maximum run number

    :   **Returns:**: the maximum run number

---


### getSetNumber

```
public int getSetNumber()
```

:   Get the set number (ie which fold this is)

    :   **Returns:**: the set number for the training and testing data sets encapsulated in this event

---


### getMaxSetNumber

```
public int getMaxSetNumber()
```

:   Get the maximum set number (ie the total number of training
    and testing sets in the series).

    :   **Returns:**: the maximum set number

---


### setGroupIdentifier

```
public void setGroupIdentifier(long identifier)
```

---


### getGroupIdentifier

```
public long getGroupIdentifier()
```


---


|  |  |  |
| --- | --- | --- |
| **Overview** \| **Package** \| **Class** \| **Tree** \| **Deprecated** \| **Index** \| **Help** \| **Weka's home** | |  |
| **PREV CLASS**   **NEXT CLASS** | **FRAMES**    **NO FRAMES**     **All Classes** |  |
| SUMMARY: NESTED \| FIELD \| CONSTR \| METHOD | DETAIL: FIELD \| CONSTR \| METHOD |  |


---
